# Physician’s Guide to Arthropods of Medical Importance, 5th Edition

**DOI:** 10.3201/eid1309.070706

**Published:** 2007-09

**Authors:** Jerome Goddard, Ling Zhou

**Affiliations:** * Centers for Disease Control and Prevention, Atlanta, Georgia, USA

**Keywords:** Arthropodborne, emergence, reemergence, treatment, book review

The 21st century is well under way, and along with the new century has come the emergence and reemergence of arthropodborne diseases. Because of the growing number of challenges these diseases pose to human health, arthropods have garnered heightened concern in the medical community. The concept of symptomatic treatment at the local level may no longer be sufficient to treat certain major types of arthropod-transmitted diseases, such as West Nile virus encephalitis. Contemporary information is a critical element of primary healthcare, but specific therapy has yet to be recommended for most arthropodborne diseases. In the primary care setting, relating the arthropodborne disease to the type of lesion (e.g., bite or sting) is important, especially if vigorous supportive measures would be helpful. Now, more than ever, raising awareness, knowledge, and treatment competency of the healthcare workforce are needed so that these diseases can be quickly diagnosed and effectively managed.

The fifth edition of Physician’s Guide to Arthropods of Medical Importance has been thoroughly updated to ensure authoritative coverage of this complex and fast-moving discipline of arthropodborne diseases, and serves as a reliable reference covering all of the major aspects of entomology. Throughout the book, the author’s vast experience and dedication are evident. The uniqueness of this book lies in its portrayal of all arthropod pests, with and without human impact, and in its emphasis on arthropods as the cause of disease. A specific arthropod is described in each section, which begins with illustrations for identification, life cycle drawings (if applicable), and succinct text regarding the arthropod’s general importance, geographic distribution, and behavior. The text also covers all harmful effects on human health and highlights current management recommendations. Written in a clear and readable style, the text presents complex, academic information on the biodiversity of arthropod pests that even a lay person can understand. As such, it is an excellent resource for clinicians, public health workers, and the public. Educational awareness of signs and symptoms of arthropodborne diseases will reduce unnecessary panic in the event of outbreaks and assist clinicians in differential diagnosis and treatment. This guide, an interdisciplinary contribution to the field of vectorborne diseases, bridges entomology, primary care, and public health.

The following minor changes could be incorporated in the next edition of this outstanding text: 1) on page 7, the end of the second paragraph should be “... oxygen free radicals and several proteins ...”; 2) the CD-ROM included has technical problems—under BugCoach, none of the 4 interactive links (Bugs, Conditions, Useful Links, Identification Helps) can be accessed successfully; and 3) in Chapter 9 on signs and symptoms of arthropodborne disease, photographs would help clinicians with diagnosis and proper treatment and the public about when or if to seek medical attention. The author and the publishers are highly commended for presenting such a comprehensive, highly useful volume on the status of arthropods in current medical concerns.

